# Knowledge, Attitudes and Practice of Iranian Patients on Sunscreen Application

**DOI:** 10.1111/jocd.70598

**Published:** 2025-12-11

**Authors:** Taraneh Yazdanparast, Mansour Nassiri Kashani, Sina Babakhani, Saman Ahmad Nasrollahi, Fatemeh Amiri, Martin Kassir, Alireza Firooz

**Affiliations:** ^1^ Center for Research and Training in Skin Diseases and Leprosy Tehran University of Medical Sciences Tehran Iran; ^2^ World Laser Institute Dallas Texas USA

**Keywords:** dermatology, patient knowledge, photoprotection, skin cancer, sunscreen

## Abstract

**Background:**

Exposure to ultraviolet (UV) radiation is a major risk factor for photoaging and skin cancer. Use of sunscreens is an essential preventive strategy, but its efficacy is hampered by incorrect application and low knowledge.

**Aims:**

The purpose of this study was to determine the parameters linked to appropriate photoprotection behaviors and to evaluate the general public's knowledge, attitudes, and practices surrounding the usage of sunscreen.

**Methods:**

A cross‐sectional study was conducted in Tehran, Iran, on 292 patients referred to two private skin clinics. They were asked to complete a questionnaire measuring the participants’ attitudes and knowledge regarding the usage of sunscreen based on the American Academy of Dermatology recommendations. Clinical and demographic information was also gathered. Associations between patient features, attitudes, and knowledge were investigated by statistical analysis.

**Results:**

Although 61.5% of participants agreed that using sunscreen every day is essential, there were clear knowledge gaps on the minimum sun protection factor (SPF) recommendations (33.8%), when to apply it (33.3%), and how long to wait between applications (38.1%). Price had an impact on attitudes about sunscreen; 34.5% of respondents said they would use it more often if it were given away for free. Information on sunscreen was primarily obtained from dermatologists (47.3%). Knowledge and variables, including age, gender, and educational attainment, were shown to be significantly correlated (*p* < 0.05).

**Conclusion:**

Even though most people understand how important sunscreen is, there is still important information gaps that might result in insufficient UV protection. Enhancing sunscreen adherence through public health campaigns and targeted education by healthcare professionals is essential to reduce skin cancer risk.

## 1 Introduction

High levels of ultraviolet (UV) radiation resulting from excessive sun exposure cause numerous acute and chronic harmful effects on the skin [1]. Both UV and visible light have significant biological effects on the skin. Visible light can induce erythema in individuals with fair skin and pigmentation in those with darker skin. Moreover, it dramatically increases the skin's production of natural pigments and reactive oxygen species [2]. Tinted sunscreens use different formulations and concentrations of iron oxide and tinted titanium dioxide to offer protection against visible light, with the best results in the blue‐light spectrum; however, the degree of protection diminishes with increasing wavelength [3, 4].

Another known and controllable risk factor for melanoma, basal cell carcinoma (BCC), squamous cell carcinoma (SCC), and other skin cancers is exposure to UV light [5]. Skin cancer (SC) is the fifth most prevalent type of cancer and one of the deadliest malignancies of the current decade [6]. It is estimated that 90% of SCCs and BCCs and 67% of melanomas are attributed to excessive sun exposure [7]. The development of BCC is primarily associated with intense UV exposure during childhood and adolescence, whereas SCC is linked to chronic, and cumulative UV exposure over decades [8]. By taking precautions, the negative effects of sun exposure can be lessened. Sun‐protective practices—such as seeking shade, avoiding tanning beds, wearing protective clothes, using sunscreen, and limiting sun exposure between 10 am and 2 pm, are beneficial in minimizing sun‐induced effects [9, 10]. According to research, SPF and broad‐spectrum UVA/UVB protection are the most crucial factors for people to consider when choosing a sunscreen, as well as the sunscreens that specialists advise using [11].

Applying sunscreen with a sun protection factor (SPF) of at least 30, 15 min before going outside and reapplying it every 2 h is advised by the American Academy of Dermatology (AAD) [12]. A minimum SPF of 30 is suggested because individuals tend to apply sunscreen in smaller amounts than required, and a higher recommended SPF compensates for under‐application. Proper sunscreen use protects against sunburn and photoaging and has been shown to minimize the incidence of skin malignancies [13, 14]. Despite the significance of using sunscreen correctly, research from all around the world shows that poor application and a lack of understanding are widespread [15, 16]. Additionally, prior research has demonstrated that Iranians use sunscreen at low rates. Even while some people know a lot about sun protection, their attitudes and behaviors have not been up to par [17, 18].

Currently, there are limited studies evaluating sunscreen use among dermatologic patients, particularly in the Iranian population with skin types II–IV. Tehran, the capital city where the study was conducted, generally experiences a UV index ranging from 3 to 6 in winter and 8 to 11 in summer, resulting in a moderate to very high risk of sun exposure for residents all year‐round [19]. This study aims to evaluate the knowledge and attitudes of dermatologic patients in the Iranian population with skin types II–IV regarding sunscreen use.

## 2 Material and Methods

### 2.1 Study Design and Setting

This cross‐sectional study was conducted at two private skin clinics in Tehran, Iran. Patients who visited these clinics were asked to fill out a structured questionnaire that evaluated their attitudes and knowledge on the usage of sunscreen, in accordance with the AADs recommendations.

### 2.2 Participants

The study included patients with dermatological disorders who visited the study clinics, were between the ages of 15 and 70 years, were willing to participate, gave their informed consent, and were available for follow‐up if needed. People who had a known allergy to sunscreen were excluded. The study's objectives determined the sample size in order to guarantee representativeness, and a convenience sampling technique was used to choose participants. All eligible and interested individuals were enrolled.

### 2.3 Data Collection

Participants' attitudes and knowledge about wearing sunscreen were evaluated using a standardized questionnaire. The survey was modified from the Vasicek et al. study [20]. The AAD sunscreen application instructions served as the basis for the knowledge exam. The questionnaire comprised three sections: Six knowledge‐based questions that address topics including SPF requirements, how often to reapply sunscreen, and how to use it depending on the situation; three questions about attitudes, assessing opinions about the efficacy of sunscreen, readiness to apply sunscreen frequently, and confidence in expert advice; and one question that asked whether patients relied on doctors, the media, or other sources for information about using sunscreen.

Along with these inquiries, clinical information (Fitzpatrick skin type, reason for clinic visit, history of personal or family skin cancer, and sunscreen allergy) and demographic information (age, gender, and educational attainment) was gathered.

### 2.4 Study Procedures

Participants who met the eligibility criteria were approached by trained research assistants. They were given detailed information about the study objectives and asked to provide oral informed consent before completing the questionnaire. The questionnaire was self‐administered, but assistance was provided upon request. Data collection was monitored to ensure completeness and accuracy.

### 2.5 Statistical Analysis

SPSS version 20 was used for data analysis. Descriptive statistics were presented, including frequencies and percentages for categorical variables and means and standard deviations for continuous data. The chi‐square test was used to compare categorical variables. Subgroup analyses were also performed to look at any variations according to Fitzpatrick skin type, age, gender, education level, and skin cancer history. Statistical significance was defined as a significance level of *p* < 0.05.

## 3 Results

The study included 292 participants, with a mean age of 39.34 years (SD = 15.47) and a gender distribution of 15.8% male and 84.2% female. Participants’ ages ranged from 15 to 70 years, indicating a broad but representative age distribution of dermatology patients. Fitzpatrick skin types III (55.1%) and IV (36.3%) were present in the majority. 98.6% of the subjects had no history of skin cancer, and 92.5% were not sensitive to sunscreen. In terms of education, 20.5% had a master's degree, 36.3% had a bachelor's degree, and 24.0% had only completed high school (Table 1). The reasons for patients’ visiting the dermatology clinic included the following, in order of prevalence: non‐photosensitive diseases (59.5%), photosensitive inflammatory diseases (22.6%), pigmentary disorders (10.7%), and others (7.1%).

**TABLE 1:** Demographic and clinical characteristics of patients.

Demographics		
Patient status	Number	Percentage
**Sex**		
Male	46	15.8
Female	246	84.2
**Previous skin cancer**		
Yes	4	1.4
No	288	98.6
**Sensitivity to sunscreen**		
Yes	22	7.5
No	270	92.5
**Fitzpatrick skin type**		
II	25	8.6
III	161	55.1
IV	106	36.3
**Educational level**		
Under high school diploma	20	6.8
High school diploma	70	24.0
Associate degree	22	7.5
Bachelor's degree	106	36.3
Master's degree	60	20.5
Doctorate	14	4.8
**Age**		
≤ 40 years	172	58.9
≥ 41 years	120	41.1
Mean	SD	
39.34	15.47	

According to the AAD recommendations, 61.5% of respondents correctly identified the need for daily, year‐round sunscreen application. Only 33.8% of respondents were aware that 30 is the minimum advised SPF, and only 9.3% were aware that 30 mL of sunscreen is required to completely cover the body before going outside. In terms of when to apply sunscreen, 33.3% of respondents correctly said that it should be applied at least 15 min before going outside, and 38.1% knew that it should be reapplied every 2 h. Furthermore, 75.0% correctly identified 10 am–4 pm as the worst time for sun exposure (Table 2).

**TABLE 2:** Patients’ knowledge of sunscreen use guidelines based on the AAD.

How often should you wear sunscreen, according to the AAD?						
With anticipated prolonged sun exposure	Daily during the summer		Daily year‐round^a^	Never	Do not know	
19.8%	13.2%		61.5%	3.1%	2.4%	
**What is the minimum number SPF that the AAD recommend people wear daily?**						
SPF 15	SPF 30^a^	SPF 45	SPF 50	None	Do not know	
1.4%	33.8%	4.3%	31.3%	0.7%	28.4%	
**How much sunscreen (in mL) should be used to cover the entire body?**						
15 mL	30 mL^**a**^	8 mL	more than 60 mL	None	Do not know	
9.3%	9.3%	3.6%	2.5%	3.2%	72.1%	
**What is the minimal amount of time (in minutes) before sun exposure you should apply sunscreen?**						
0 min	15 min^**a**^	30 min	45 min	60 min	Do not know	
7.2%	33.3%	26.2%	2.5%	7.2%	23.7%	
**What is the longest amount of time (in hours) that should be allowed between of sunscreen?**						
1 h	2 h^**a**^	3 h	4 h	6 h	Never	Do not know
2.9%	38.1%	16.9%	15.8%	8.6%	1.4%	16.2%
**What is the worst time to be exposed to the sun?**						
Before sunset	8 a.m.–10 a.m.		10 a.m.–4 p.m.^a^		Do not know	
5.4%	7.1%		75.0%		12.5%	

Abbreviations: AAD, American academy of dermatology; SPF, sun protection factor.

^**a**^ The AAD recommendation for the category in question.

When assessing attitudes toward sunscreen use, 47.0% of participants reported that sunscreen price did not impact their choice, and 72.4% indicated that price did not prevent them from purchasing it. Furthermore, 34.5% of participants said that receiving free sunscreen from the hospital would encourage them to use it more frequently (Table 3).

**TABLE 3:** Patients’ attitudes toward sunscreen use based on the AAD.

Does the price of sunscreen impact your sunscreen choice?		
I do not know	No	Yes
7.5%	47.0%	45.5%
**Does the price of sunscreen prevent you from buying it?**		
I do not know	No	Yes
6.5%	72.4%	21.1%
**Will receiving free sunscreen from the hospital increase the possibility of you using sunscreen regularly?**		
I do not know	No	Yes
18.3%	34.5%	47.1%

**FIGURE 1:**
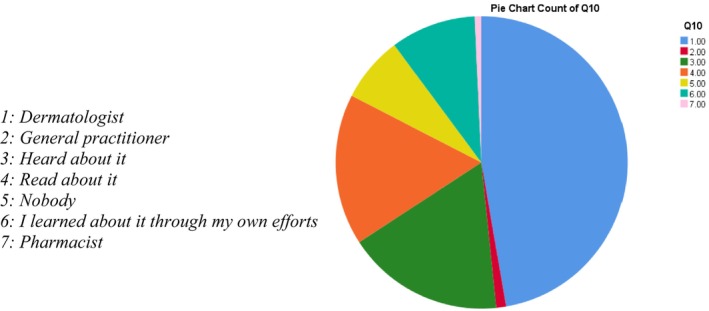
Source of information for dermatological patients on the correct way to use sunscreen.

Dermatologists were the most often cited source of information regarding sunscreen (47.3%), followed by individual listening (17.5%), individual reading (16.7%), and independent research (9.5%). Only 7.3% of respondents said they had no information source. The sources of information for patients were 1.1% and 0.7% of general practitioners and pharmacists, respectively (Figure 1).

Significant associations were found between knowledge and demographic variables. Gender was significantly related to knowledge about the longest interval for sunscreen reapplication; females were more aware (40.4% vs. 25.6%, *p* = 0.039), and age was significantly associated with knowledge about the worst time for sun exposure. People over 40 years were more aware than those who were 40 years or younger (75.9% vs. 74.4%, *p* < 0.0001). Educational level was correlated with knowledge of the minimum recommended SPF and the correct time of applying sunscreen before sun exposure. People who had a Bachelor's degree were more aware than other educational levels about the minimum recommended SPF (*p* = 0.009), and people who had a PhD degree were more aware than other educational levels about the correct time of applying sunscreen before sun exposure (*p* = 0.048). Additionally, knowledge of the worst time for sun exposure was significantly associated with having a history of specific skin disease; people who had a history of pigmentary disorders were more aware than others (*p* = 0.007) (Table 4).

**TABLE 4:** Association between knowledge of proper sunscreen use and other variables among study participants.

	Frequency of use	Minimum SPF	Amount needed	Time before exposure to sun	Time before reapplication	Worst time of exposure
**Gender**	*p* = 0.084	*p* = 0.066	*p* = 0.056	*p* = 0.676	*p = 0.039*	*p* = 0.545
Females	65.0%	36.0%	9.3%	32.3%	40.4%	75.0%
Males	42.2%	21.4%	9.1%	38.6%	25.6%	75.0%
**Age**	*p* = 0.149	*p* = 0.334	*p* = 0.829	*p* = 0.548	*p* = 0.552	*p* < 0.0001
40 years or younger	60.6%	32.7%	9.6%	35.6%	41.1%	74.4%
Over 40 years	62.7%	35.4%	8.8%	30.2%	33.9%	75.9%
**Educational level**	*p* = 0.306	*p = 0.009*	*p* = 0 0.087	*p = 0.048*	*p* = 0.067	*p* = 0.068
Less than high school	50.0%	26.3%	21.1%	10.0%	35.0%	70.0%
High school	59.4%	26.6%	6.0%	33.3%	27.3%	72.7%
Associate degree	50.0%	28.6%	14.3%	33.3%	40.0%	90.5%
Bachelor's degree	64.1%	39.6%	7.9%	36.4%	42.4%	71.0%
Master's degree	65.0%	35.0%	11.7%	33.9%	44.1%	81.4%
PhD	71.4%	38.5%	0.0%	42.9%	35.7%	71.4%
**Fitzpatrick skin type**	*p* = 0.893	*p* = 0.570	*p* = 0.476	*p* = 0.858	*p* = 0.714	*p* = 0.837
II	66.7%	45.8%	13.0%	18.2%	36.4%	73.9%
III	60.4%	32.7%	8.6%	34.6%	40.5%	77.1%
IV	61.9%	32.7%	9.5%	34.6%	35.0%	72.1%
**Skin disease**	*p* = 0.774	*p* = 0.689	*p* = 0.095	*p* = 0.606	*p* = 0.422	*p* = 0.007
Photosensitive inflammatory diseases	64.3%	35.2%	14.3%	28.6%	34.5%	75.0%
Pigmentary disorders	66.7%	33.3%	15.4%	26.9%	57.7%	84.6%
Non‐photosensitive diseases	55.4%	33.3%	5.6%	38.1%	36.0%	72.1%
Others	72.2%	17.6%	11.8%	33.3%	44.4%	83.3%
**Previous skin cancer**	*p* = 0.510	*p* = 0.141	*p* = 0.978	*p* = 0.111	*p* = 0.465	*p* = 0.866
Yes	25.0%	25.0%	25.0%	25.0%	25.0%	100.0%
No	62.0%	33.9%	9.1%	33.5%	38.3%	74.6%

*Note:* All *p*‐values are typed italic to differentiate from other numbers.

Regarding attitudes, there was a significant correlation between educational attainment and the influence of sunscreen price on buying behavior (*p* = 0.045). People who had a PhD degree showed the most inhibitory effect of sunscreen price on buying it (28.6%). There was also a significant correlation between the likelihood of regularly using sunscreen when it was free and having photosensitive inflammatory diseases (63.6%, *p* < 0.0001). There was a significant correlation between the likelihood of regularly using sunscreen when it was free and having no prior skin cancer (47.6%, *p* = 0.040) (Table 5).

**TABLE 5:** The relationship between the attitude of study participants regarding the use of sunscreen and other variables.

	Sunscreen price effect on choosing sunscreen	Inhibitory effect of sunscreen price on buying it	Increasing sunscreen use due to receiving free sunscreen
Gender	*p* = 0.163	*p* = 0.251	*p* = 0.719
Females	43.8%	20.0%	46.6%
Males	54.5%	27.3%	50.0%
Age	*p* = 0.834	*p* = 0.991	*p* = 0.254
40 years or younger	47.2%	22.1%	49.7%
Over 40 years	43.1%	19.8%	43.5%
Educational level	*p* = 0.732	*p = 0.045*	*p* = 0.621
Less than high school	45.0%	5.3%	50.0%
High school	50.0%	22.7%	54.5%
Associate degree	38.1%	9.5%	33.3%
Bachelor's degree	41.4%	22.0%	47.0%
Master's degree	49.2%	25.4%	47.5%
PhD	50.0%	28.6%	28.6%
Fitzpatrick skin type	*p* = 0.661	*p* = 0.243	*p* = 0.764
II	47.8%	13.6%	60.9%
III	46.7%	19.0%	43.0%
IV	43.3%	26.0%	50.0%
Skin disease	*p* = 0.388	*p* = 0.903	*p* < 0.0001
Photosensitive inflammatory diseases	53.6%	21.8%	63.6%
Pigmentary disorders	38.5%	15.4%	53.8%
Non‐photosensitive diseases	45.7%	23.6%	44.6%
Others	29.4%	5.6%	22.2%
Previous skin cancer	*p* = 0.698	*p* = 0.272	*p = 0.040*
Yes	50.0%	0.0%	0.0%
No	45.5%	21.5%	47.6%

## 4 Discussion

The study's findings showed that dermatological patients had a substantial knowledge gap on how to properly apply sunscreen. When asked about the best times to be in the sun and when to apply sunscreen, more than half of the participants were able to provide accurate answers. Less than half of the participants, however, were aware of the lowest SPF that is advised, the shortest amount of time before going outside to apply sunscreen, the longest period between applications, and the appropriate quantity of sunscreen needed to cover the full body. Only a small proportion knew these important facts. These results imply that there is a lack of knowledge on important sunscreen application factors, which might lead to inadequate sun protection and a higher risk of skin damage and skin cancer. In Iran, non‐melanoma skin cancers are among the most common malignancies, and their rising incidence may be partly related to insufficient photoprotection and knowledge gaps such as those observed in our study [21].

Although similar studies have been conducted in other regions, the novelty of our research stems from its emphasis on dermatology patients in Iran, a demographic frequently neglected in earlier investigations. Their cultural habits, healthcare access, and frequent contact with dermatologists create a distinctive setting, and employing a standardized questionnaire enables significant comparison with global results [22, 23].

Dermatologists were the most often cited source of information for patients seeking advice on how to apply sunscreen, while pharmacists received the fewest mentions. This is consistent with research in the literature, which shows that dermatologists often treat skin‐related issues, such as preventing photodamage and skin cancer [20]. Furthermore, it has been demonstrated that using sunscreen correctly lowers the incidence of UV‐induced skin lesions. The main rules are to apply a suitable amount of sunscreen at least half an hour before going outside and to reapply it every 2 h [16, 24]. According to a US survey, the majority of people did not receive enough advice on how to apply sunscreen and did not fully understand its directions [25]. We focused on dermatology patients because they differ from community samples by having more frequent medical contact and higher awareness of skin health. Studying this group provides clinically relevant insights and complements findings from general population studies [26].

In terms of attitudes, most participants (72.4%) reported that the price of sunscreen did not prevent them from purchasing it, although nearly half acknowledged that cost influenced their choice, and about one‐third indicated they would use sunscreen more frequently if it were provided for free. Although broad‐spectrum sunscreen is readily available, little is known about how patients feel about sunscreen recommendations or how well they comprehend the advice of medical professionals [27].

Additionally, certain knowledge‐related questions and characteristics, including age, gender, education level, and history of skin illnesses, were shown to be statistically significantly correlated. Similarly, educational attainment, skin disease history, and prior skin cancer diagnosis all had an impact on views toward sunscreen [28]. These results are in line with a Michigan, USA study that discovered that skin type and gender had an impact on sunscreen use knowledge. The study underlined the significance of offering additional counseling and instruction on appropriate sunscreen use in order to achieve effective primary prevention of skin cancer [20]. Higher education was associated with better knowledge in some domains, but this relationship was not consistent across all aspects of sunscreen use. This finding has been shown about knowledge about some other subjects which were so important for special patients [24]. Although our study did not find a strong association between skin type and sunscreen knowledge, previous research suggests that individuals with darker skin types may perceive themselves as less vulnerable to UV damage, potentially leading to inadequate photoprotection and a higher cumulative risk of skin cancer.

In our research, most of the participants were female (84.2%). This gender disparity might be associated with cultural and social factors in Iran, where women are more likely to visit dermatology clinics due to greater concern for skin appearance, beauty ideals, and sun protection [29]. In contrast, men tend to participate less in preventive skin care, likely due to existing cultural norms surrounding masculinity [30]. Previous studies have shown that masculine norms can act as barriers to sunscreen use among men, who may perceive sun protection behaviors as less aligned with masculine identity [31]. Additionally, studies show that men typically report using less sunscreen than women, with various factors such as lack of knowledge and views on skin care contributing to this difference [32, 33]. This cultural aspect might partly account for the higher number of females in our study sample and underscores the necessity for gender‐specific public health initiatives to enhance sunscreen adherence [34].

Similar research conducted in Germany (2018) showed that while 79.4% of participants used sunscreen at least sometimes, 87.2% did not apply it at the required time, and 59.5% did not reapply it as directed. Regardless of skin type, the study found a general lack of compliance with health authorities' recommendations for sunscreen use, highlighting the necessity of focused public education efforts or one‐on‐one medical counseling [16].

In comparing our findings with recent international studies, several similarities can be noticed. Lim et al. (2024) reported that although sunscreen application was fairly frequent in 20 countries, only about one‐quarter of participants reapplied it every 2 h, indicating significant gaps in proper usage [35]. Similarly, Hafez et al. (2024) showed that while sunscreen use was prevalent in Saudi Arabia, knowledge about recommended SPF and daily application was still insufficient [36]. Consistent with these results, our participants were aware of the general importance of sunscreen but demonstrated substantial knowledge gaps regarding SPF, timing, and reapplication. Glanz et al. (2022) also highlighted that even in high‐resource settings, adults often misunderstand sunscreen guidelines and show inconsistent adherence [37]. Together, these findings suggest that inadequate knowledge and improper application of sunscreen are global issues, underscoring the need for culturally adapted education approaches.

The majority of participants in comparable research conducted in Saudi Arabia had heard of sunscreen (93.4%) and had used it previously (72%), but there were still knowledge gaps, especially when it came to the required SPF and daily usage of sunscreen. Just 13.5% of participants were aware of the minimum required SPF, and only 20.5% were aware that sunscreen should be applied every day of the year. Just 4.5% of participants recognized the appropriate amount of sunscreen required for full body coverage, while a sizable majority (34%) did not know the minimum length of time before sun exposure for sunscreen application [12].

Cultural practices also need to be considered when interpreting our findings. In Iran, intentional sunbathing is rare, and traditional clothing often reduces direct sun exposure, particularly among women [17]. Nevertheless, sunscreen use was still inadequate, suggesting that beyond cultural habits, gaps in knowledge and low prioritization of sunscreen also play a role [38]. These factors highlight the importance of tailoring photoprotection strategies to local cultural and lifestyle contexts.

## 5 Limitations of the Study

This study has some limitations even if it offers insightful information on dermatological patients’ attitudes and knowledge regarding sunscreen use. First, the study was cross‐sectional, which means that causal linkages between variables cannot be established because it only records data at one particular moment in time. Second, the findings’ generalizability may be limited by the sample size's potential lack of representativeness of the larger group of dermatological patients. Furthermore, because the study used self‐reported data, individuals could have inflated their knowledge or behavior about the usage of sunscreen, which might have created bias. Additionally, the study did not take into consideration participant differences in sunscreen kinds, which may have influenced their experiences and understanding.

## 6 Conclusion

This study shows that although dermatology patients generally understand the value of using sunscreen, their understanding of particular recommendations—such as the right amount of sunscreen, the right SPF, when to apply it, and how often to reapply it—remains inadequate. Furthermore, although opinions on the usage of sunscreen were generally favorable, knowledge and opinions were strongly impacted by variables including gender, age, education, and a history of skin conditions. Giving patients thorough instruction and counseling on how to apply sunscreen is essential for the primary prevention of skin cancer and photodamage. The dissemination of this knowledge is mostly the responsibility of healthcare professionals, especially dermatologists. To increase public awareness and compliance with sunscreen recommendations, public health campaigns and focused educational initiatives are required. Practical measures may include incorporating photoprotection counseling into routine dermatology care, promoting nationwide awareness campaigns, and utilizing social media platforms to engage younger audiences.

## Author Contributions

M.N.K., M.K., and A.F. designed the research study. T.Y., S.A.N., and F.A. performed the research study. F.A. and A.F. contributed essential reagents or tools. T.Y., S.B., and S.A.N. analyzed the data. T.Y., S.B., and A.F. wrote the research paper. M.N.K. and M.K. edited the research paper.

## Funding

The authors have nothing to report.

## Ethics Statement

This study was approved by the Ethics Committee of Tehran University of Medical Sciences (ethics code: IR.TUMS.MEDICINE.REC.1401.822) and approval of the study proposal has been obtained by the Center for Research and Training in Skin Diseases and Leprosy.

## Consent

Written informed consent was obtained from all participants prior to participation in the study.

## Conflicts of Interest

The authors declare no conflicts of interest.

## Data Availability

The data that support the findings of this study are available on request from the corresponding author. The data are not publicly available due to privacy or ethical restrictions.
